# Secondary effects after belantamab treatment: A new anti‐B‐cell maturation antigen monoclonal antibody for multiple myeloma

**DOI:** 10.1002/jha2.205

**Published:** 2021-05-07

**Authors:** Sara Garrido Paniagua, Pablo Prieto Martínez, Rafael Forés Cachón, Guiomar Bautista Carrascosa, Rafael F. Duarte, Isabel Krsnik

**Affiliations:** ^1^ Hematology Department Hospital U. Puerta de Hierro‐Majadahonda Majadahonda Madrid 28222 Spain

We present the case of a 59‐year‐old male with an IgG multiple myeloma diagnosed in 2014. The patient had received since then, therapy with a bortezomib–thalidomide induction and a first autologous stem cell transplantation (SCT) followed by maintenance with lenalidomide and Ixazomib, never obtaining a complete response. He relapsed in 2019 and was treated with daratumumab without response. He received a course of standard chemotherapy achieving a very good partial response, followed by a second autologous SCT in February 2020.

The patient was admitted in May 2020 in clinical and biochemical progression, with a Horner syndrome due to a thoracic plasmacytoma. After reviewing previous therapy, we decided to use belantamab, a new anti‐BCMA (B‐cell maturation antigen) monoclonal antibody on a compassionate basis. Four days after the first dose, a sudden worsening of cytopenias and increase in acute phase reactants were observed: 1.2 × 10^−3^ /microL leukocytes (4.0–11.5), 0.8 × 10^−3^ /microL neutrophils (1.5–7.5), 0.16 × 10^−3^ /microL lymphocytes (1.2–4.0), 7 g/dL hemoglobin (12.0–17.0), 10 × 10^−3^ /microL platelets (150.0–400.0). Ferritin 15,216 ng/mL (30.0–300.0) and lactate dehydrogenase (LDH) 2697 U/L (120.0–246.0).

**FIGURE 1:**
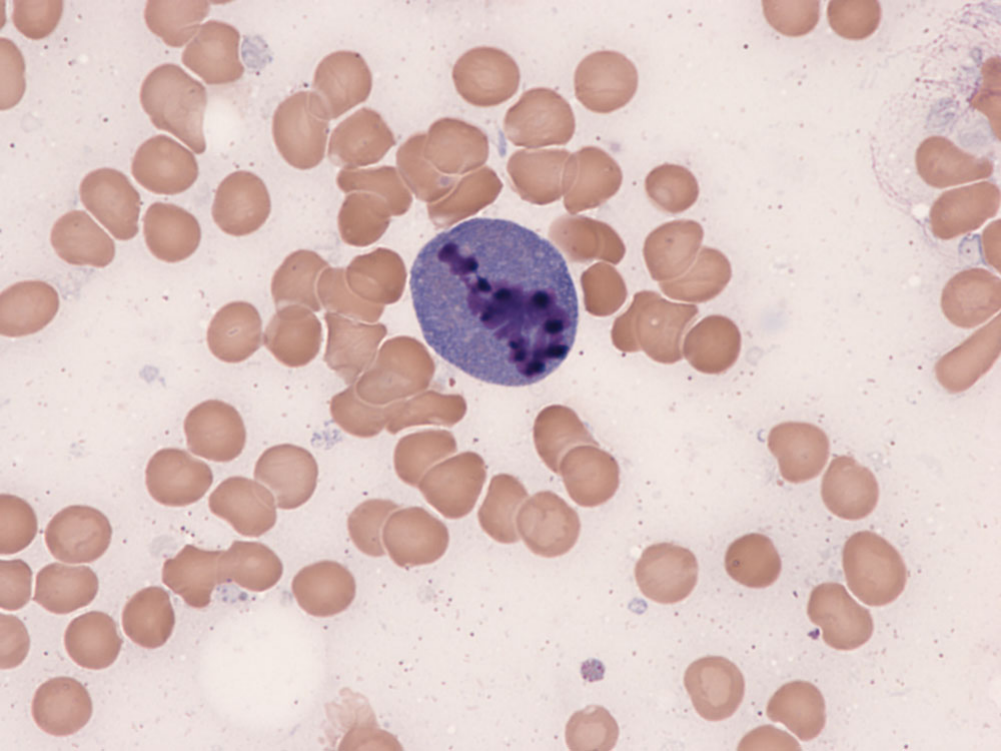
Bone marrow smear, wright staining, 500 X. It can be seen one plasma cells with nucleus in apoptosis and simultaneously, a macrophage phagocytosing cellular debris from other plasma cells

**FIGURE 2:**
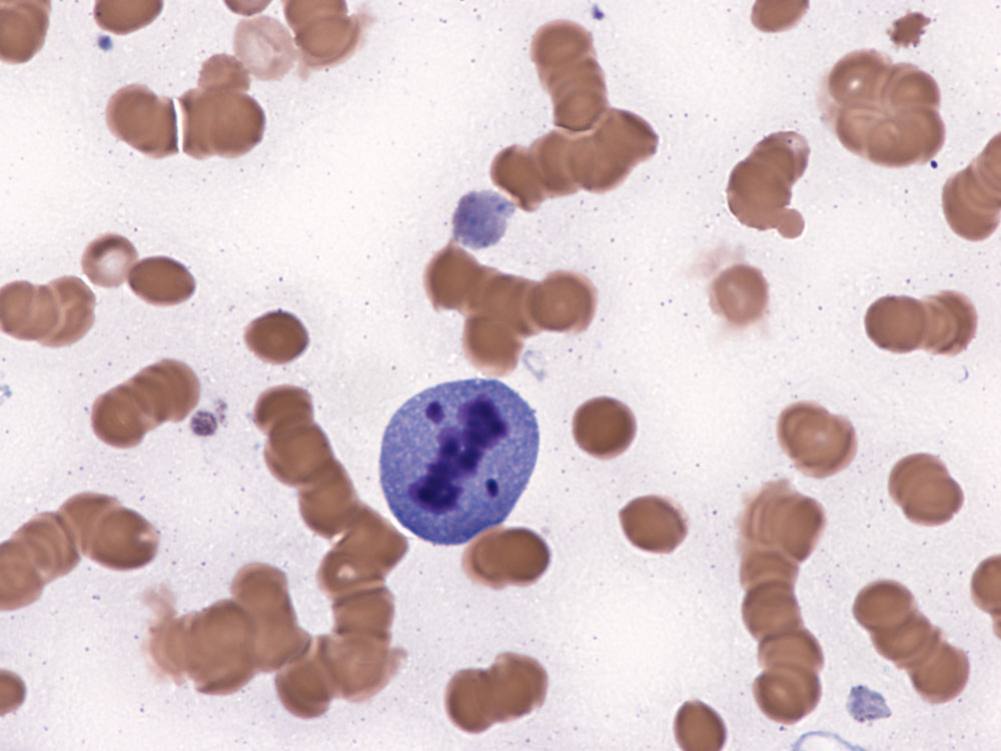
Bone marrow smear, Wright staining, 1000X. Large plasma cell with an apoptotic nucleus

**FIGURE 3:**
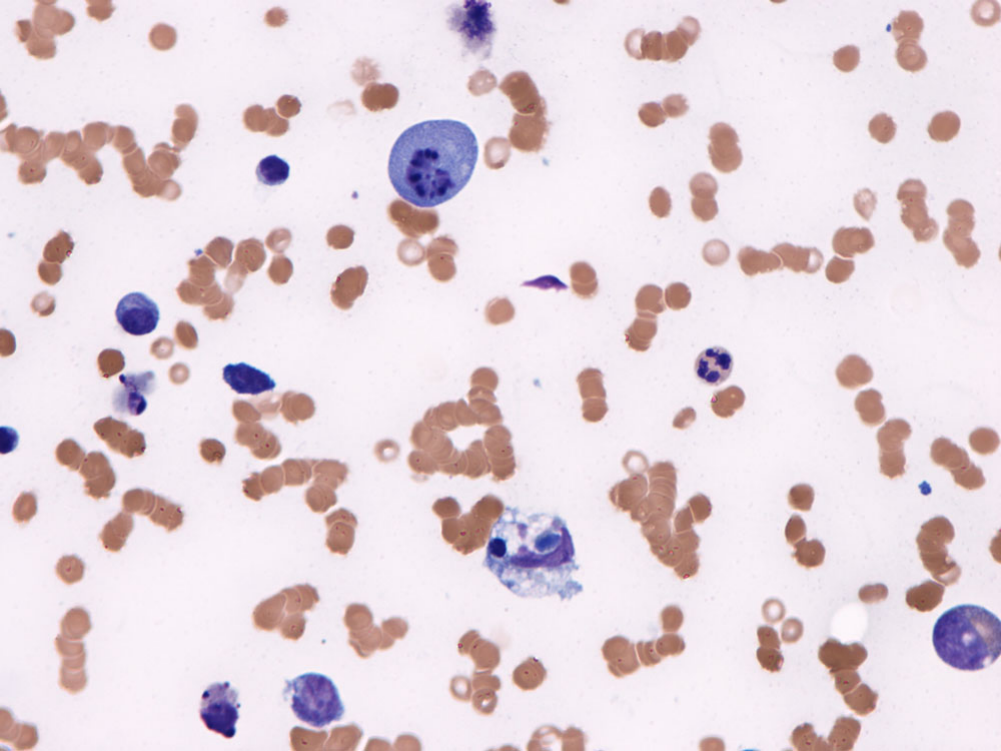
Bone marrow smear, Wright staining, 1000X. Large plasma cell with an apoptotic nucleus

**FIGURE 4:**
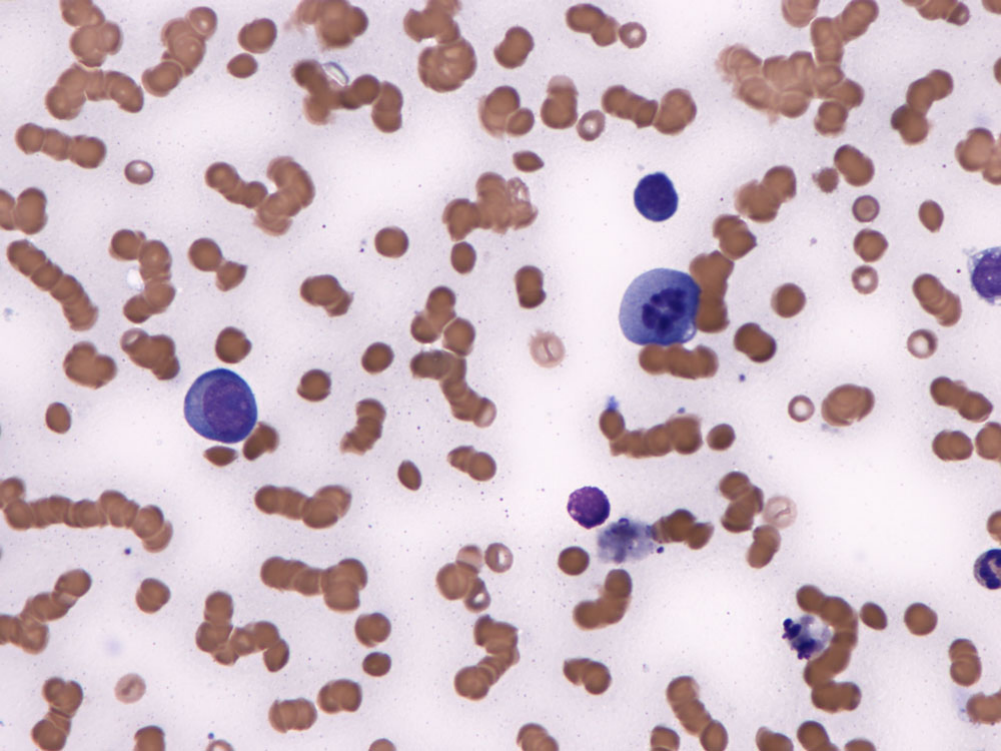
Bone marrow smear, wright staining, 500 X. It can be seen one plasma cells with nucleus in apoptosis and simultaneously, a macrophage phagocytosing cellular debris from other plasma cells

A bone marrow aspirate was performed to rule out hemophagocytic syndrome (HPS), since he also had fever and met criteria for HPS. The aspirate showed no evidence of erythophagocytosis but it showed a striking number of plasma cells in apoptosis (Figures 1, 2), macrophage proliferation with phagocytosis of cellular debris, and isolated residual plasmablasts (Figures 3, 4), all probably due to the mechanism of action of the drug. This report highlights the presence of plasma cell apoptosis in a patient treated with this new drug, showing its action mechanism (cellular apoptosis) as opposed to the necrosis conventionally produced by chemotherapy.

